# Bioactivity Determination of a Therapeutic Recombinant Human Keratinocyte Growth Factor by a Validated Cell-based Bioassay

**DOI:** 10.3390/molecules24040699

**Published:** 2019-02-15

**Authors:** Wenrong Yao, Ying Guo, Xi Qin, Lei Yu, Xinchang Shi, Lan Liu, Yong Zhou, Jinpan Hu, Chunming Rao, Junzhi Wang

**Affiliations:** 1 National Institutes for Food and Drug Control, No. 2, Tiantan Xili, Dongcheng District, Beijing 100050, China; yz1322@126.com (W.Y.); guoying@nifdc.org.cn (Y.G.); qinxi@nifdc.org.cn (X.Q.); yulei@nifdc.org.cn (L.Y.); shixc@nifdc.org.cn (X.S.); liulan@nifdc.org.cn (L.L.); zhouyong@nifdc.org.cn (Y.Z.); 13716691086@163.com (J.H.); 2 Institutes of Process Engineering, Chinese Academy of Sciences, 1 North 2nd Street, Zhongguancun, Haidian District, Beijing 100190, China

**Keywords:** rhKGF-1, rhKGF-2, bioactivity, cell-based bioassay, method validation

## Abstract

The therapeutic recombinant human keratinocyte growth factor 1 (rhKGF-1) was approved by the FDA for oral mucositis resulting from hematopoietic stem cell transplantation for hematological malignancies in 2004. However, no recommended bioassay for rhKGF-1 bioactivity has been recorded in the U.S. Pharmacopoeia. In this study, we developed an rhKGF-1-dependent bioassay for determining rhKGF-1 bioactivity based on HEK293 and HaCat cell lines that stably expressed the luciferase reporter driven by the serum response element (SRE) and human fibroblast growth factor receptor (FGFR2) IIIb. A good responsiveness to rhKGF-1 and rhKGF-2 shared by target HEK293/HaCat cell lines was demonstrated. Our stringent validation was completely focused on specificity, linearity, accuracy, precision, and robustness according to the International Council for Harmonization (ICH) Q2 (R1) guidelines, AAPS/FDA Bioanalytical Workshop and the Chinese Pharmacopoeia. We confirmed the reliability of the method in determining rhKGF bioactivity. The validated method is highly timesaving, sensitive, and simple, and is especially valuable for providing information for quality control during the manufacture, research, and development of therapeutic rhKGF.

## 1. Introduction

Keratinocyte growth factor (KGF) was originally isolated from human embryonic lung fibroblast-conditioned medium, and it is a member of the fibroblast growth factor (FGF) family [1,2]. The FGF family is composed of 23 members and classified into six subfamilies in mammals [3]. Among these, FGF7 (also called KGF-1) [1] and FGF10 (also called KGF-2) [4] belong to the FGF7 subfamily. The human *Ffg7* gene was mapped to chromosome 15q21.2, and its 582 bp open reading frame (ORF), which consisted of four exons, could encode a native 194 amino acid monomeric polypeptide with approximately 25–30 kDa [1,5,6]. The chromosomal localization of human *Fgf10* gene was in p12-p13 region of chromosome 5. The complete ORF sequence with three exons encoded a protein of 208 amino acids. The observed molecular mass of the recombinant human FGF10 expressed in *E.coli* was approximately 19 kDa [7,8]. The conserved region of human KGF-2, i.e., amino acid 60–205, had the highest homology with that of KGF-1, sharing 54% amino acid identity. Based on the evolutionary relationship, KGF-2 is closest to KGF-1. Additionally, the specificity of mitogenic activity of KGF-2 is similar to that of KGF-1 [2,7,8]. 

Given the biological functions, KGF-1 and KGF-2 are considered paracrine factors, and regulate embryonic development by binding to their specific FGF receptor 1 (FGFR1) and FGFR2. FGFR2, which is expressed exclusively on epithelial cells, is a high affinity receptor for KGF-1 and KGF-2 [9,10,11,12,13]. It must be emphasized that KGF-1 and KGF-2 preferentially activate the IIIb splice variant of FGFR2 (FGFR2 IIIb), and KGF-2 also activates the IIIb splice variant of FGFR1 (FGFR1 IIIb) [14,15]. Upon binding to their receptors, the activated FGFR1 and FGFR2 (especially FGFR2 IIIb), autophosphorylate tyrosine residues, phosphorylate the intracellular domains of other kinases, and subsequently induce mitogen-activated protein kinases (MAPKs), the phosphoinositide 3-kinase (PI3K)/AKT/mammalian target of rapamycin (mTOR) pathway, and the phospholipase Cγ (PLCγ) intracellular signaling pathway [3,16,17,18].

Through its intracellular signal transduction, KGF induces its biological activity in keratinocytes and in the development and morphogenesis of multiple epithelial cell lineages within the skin, lung, and reproductive tract [19,20]. Therefore, KGF may have potential therapeutic benefits in the growth and development of related tissues and in wound healing [21]. In 2004, palifermin, a recombinant human KGF-1 (rhKGF-1, developed by Amgen, Thousand Oaks, CA, USA), was approved by the FDA for the treatment of severe oral mucositis in adult patients receiving myeloablative radiochemotherapy for hematological malignancies and requiring autologous hematopoietic stem cell transplants [22,23]. Palifermin has further been investigated as concomitant chemotherapy for the treatment of colorectal cancers and head/neck cancers in recent years [24]. In comparison to endogenous KGF-1, palifermin is more stable due to its removal of 23 amino acids from the N-terminal end [2,25,26]. Palifermin binds to FGFR2 IIIb, thereby inducing the proliferation, differentiation, and migration of epithelial cells. It also inhibits apoptosis of epithelial cells and repairs damaged epithelium [23,27]. Nowadays, repifermin (recombinant human KGF-2) demonstrated mixed results in a clinical trial of topical treatment for healing of chronic venous leg ulcers [9].

Understanding the biological activity of therapeutic rhKGF-1 is critical for clinical safety and efficacy, especially prior to its use in humans. However, bioassays of rhKGF-1 have not been recorded in the U.S. Pharmacopoeia, although palifermin was approved for the therapeutical use. Previous studies had shown that KGF-1 promoted mitogenic activity by a [^3^H] thymidine incorporation assay in human FGFR2b-expressing BaF3 cells and type II alveolar cells [15,18,28]. Recently, the MTT assay showed that rhKGF-1 had a significant proliferative effect on the NIH3T3, A549b, and MCF7 cell lines [26]. However, the aforementioned bioassay is time-consuming and tedious (88 h–160 h per experimental procedure) and high variability with low signal-noise-ratio (SNR). Reporter gene assays (RGAs) are Mechanism of Action (MOA)-related, less variable, higher sensitive and labor-saving. They have been increasingly adopted as quality control of biopharmaceuticals [29,30].

Herein, an RGA for determining the bioactivity of therapeutic rhKGF was developed based on HEK293 and HaCat cell lines stably transfected with luciferase reporter gene controlled by the serum response element (SRE) promoter and human FGFR2 IIIb. Upon rhKGF binding to FGFR2 IIIb and subsequent intracellular signaling cascades, the interaction between the transcription factor and SRE drives downstream luciferase gene expression. The bioactivity of rhKGF was determined by measuring relative luciferase units (RLU) driven by SRE. The new method was then optimized and fully validated with respect to specificity, linearity, accuracy, and precision based on the regular requirements as stated in the (ICH) Q2 (R1) guidelines, AAPS/FDA Bioanalytical Workshop and the Chinese Pharmacopoeia. The desired results of this validation were obtained and provide invaluable information for quality control during the manufacture, research, and development of therapeutic rhKGF-1 and rhKGF-2.

## 2. Results 

### 2.1. Identification of Cells Responsive to rhKGF-1

**Figure 1:**
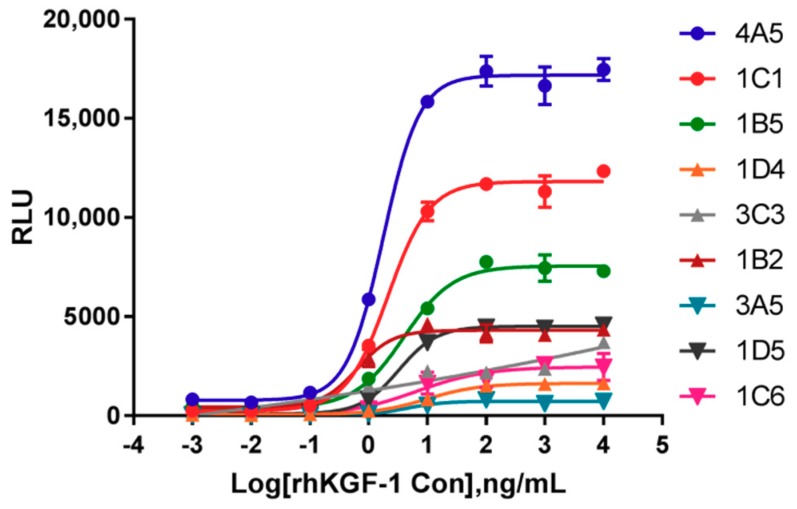
The establishment of HEK293-Luc cell lines responsive to rhKGF-1. The nine clones of single-cell dilutions of HEK293-Luc cells bearing luciferase and human KGFR2 IIIb were evaluated by their luciferase activity with rhKGF-1 stimulation (initial concentration of 10,000 ng/mL, dilution ratio of 1:10). The curves were calculated in a four-parameter model. RLU = Relative Luciferase Units. The mean ± SD is shown on each curve.

To develop responsive cell lines for rhKGF-1bioactivity, transformed HEK293 and HaCat cells bearing an SRE-luciferase reporter and human FGFR2 IIIb were constructed. By limiting dilutions, we produced nine HEK293 clonal cell lines and six HaCat clonal cell lines from single cells. All of these clones were responsive to rhKGF-1 stimulation and were hygromycin B/puromycin resistant. Of the nine HEK293-Luc clonal cell lines, a representative cell line, 1C1, was chosen and characterized in terms of its rhKGF-1-dependence. This cell line had the highest SNR, and it was more sensitive to rhKGF-1 stimulation than the other cell lines; that is, it had a lower EC_50_ value (Figure 1). Clonal cell line 1A6 from the HaCat-Luc cells was demonstrated to produce a typical sigmoidal curve much like the HEK293-Luc cell line did (Figure S1). Clones 1C1 and 1A6 were selected for further method validation.

### 2.2. Optimization Procedure

To obtain optimal sensitivity and stable results with HEK293-Luc cells, the optimal initial concentration of rhKGF-1, heparin concentration in the assay media, number of cells per well, and incubation time were investigated. Additionally, the cells were routinely cultured in DMEM-10% FBS media, which caused increased background luciferase values (Figure S2A). The SNR of 9.30 seen in this media was significantly lower than that seen in DMEM-0.5% FBS, which had an SNR of 38.07. In the experiment we designed, we optimized one parameter by changing it while keeping the others constant.

#### 2.2.1. Optimal initial rhKGF-1 concentration

**Figure 2:**
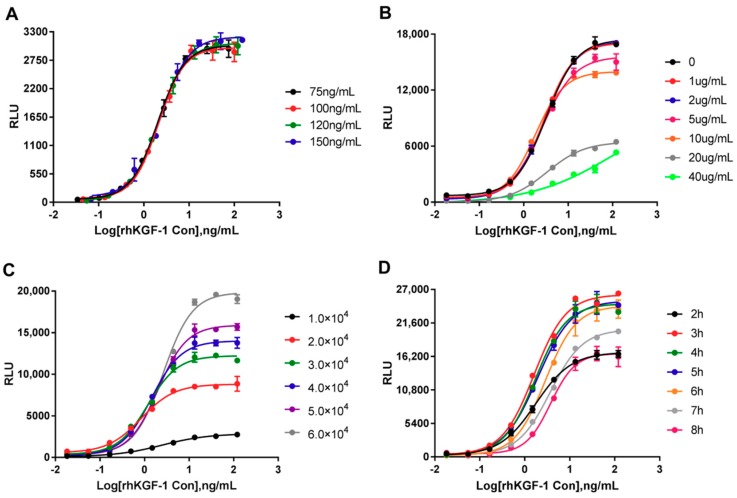
Optimization of parameters using responsive HEK293-Luc cells for determination of rhKGF-1 bioactivity. (**A**) The initial concentration of rhKGF-1. HEK293-Luc cells were stimulated at initial concentrations of 75, 100, 120 and 150 ng/mL with three dilution factors. (**B**) Heparin concentration in the assay medium. (**C**) Different numbers of HEK293-Luc cells per well added to a 96-well plate. (**D**) Incubation time after the rhKGF-1 was added to the HEK293-Luc cells. The curves were calculated in a four-parameter model. In each experiment, every dilution point was tested in three wells of cells. RLU = Relative Luciferase Units. The mean ± SD is shown on each curve.

As shown in Figure S3, luciferase activity dose-dependently increased with increasing rhKGF-1, and the sigmoidal curve drew close to its bottom asymptote and top asymptote between 0.02 ng/mL and 137 ng/mL of rhKGF-1. Thus, an optimized assay was subsequently designed, with different initial concentrations with three dilution factors. Figure 2A illustrated dose-dependent curves of luciferase activity at all of the initial concentrations. The points of the top and bottom asymptotes of 100 ng/mL and 120 ng/mL as initial concentrations met the experimental demand, although all of the curves were similar. Here, the recommended concentration in the bioassay was 120 ng/mL.

#### 2.2.2. Optimization of Heparin Concentration in Assay Media

To achieve a possibly higher sensitivity, we thought it might be necessary to supplement the assay media with heparin. We determined the suitable concentration of heparin by investigating the EC_50_ value and the SNR. As shown in Figure 2B, we found that the three curves overlapped and probably were equivalent at 0, 1 and 2 μg/mL of heparin in assay media. The RLUs at the top asymptote resulting from all but the above three concentrations were decreased gradually with increasing heparin concentrations, and the sigmoidal curves were not significant at 20 and 40 μg/mL of heparin. Based on the EC_50_ values (3.96, 3.57 and 3.58 ng/mL at 0, 1 and 2 μg/mL of heparin, respectively) and SNR’s (25.2, 35.1, and 52.7, respectively), we concluded that 2 μg/mL of heparin was superior to other heparin concentrations.

#### 2.2.3. Optimization of Cell Numbers and Incubation Time

For optimizing the number of cells per well, freshly trypsinized HEK293-Luc cells were added in various numbers to a 96-well plate. Although the RLU of the top asymptote was increased with increasing cell numbers, no significant differences in the SNR were observed. Due to the lower EC_50_ value, 4 × 10^4^ cells per well were used in the subsequent assay (Figure 2C). Finally, the highest magnitude of RLU was found between 3 h and 5 h. The curves and EC_50_ values were nearly identical for 3 h to 5 h, indicating that 3 h to 5 h was a good incubation period under the given conditions (Figure 2D). The decline in RLU between 6 h and 8 h was probably associated with a lack of rhKGF-1. Considering the ease of operation and sensitivity of the bioassay, we therefore chose a 4 h incubation as the optimal time.

### 2.3. Validation of Bioactivity Procedures

In this study, the HEK293-Luc cells responsive to rhKGF-1 were further validated according to the ICH Q2 (R1) guidelines, AAPS/FDA Bioanalytical Workshop and the Chinese Pharmacopoeia. In particular, the following typical validation parameters were considered.

#### 2.3.1. Specificity

**Figure 3:**
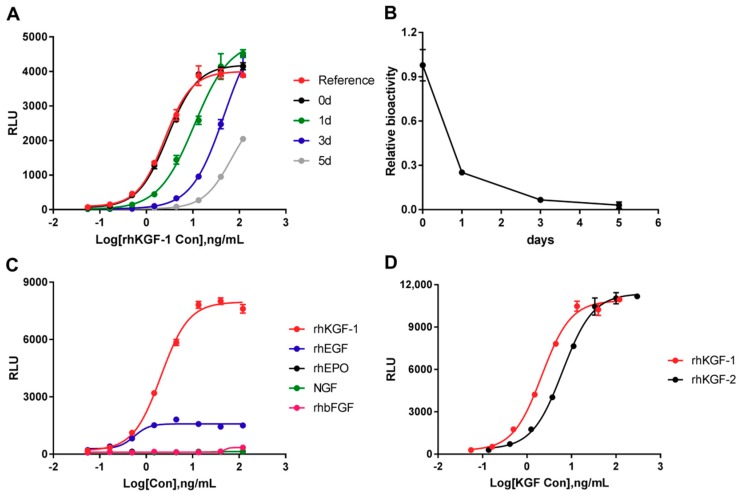
The specificity of rhKGF-1 bioassay. (**A**) The sigmoidal curves of untreated and forced degradation of rhKGF-1. The forced degradation was induced by 37 °C incubation for 1 d, 3 d and 5 d. (**B**) The relative bioactivity of forcibly degraded rhKGF-1. The relative bioactivities were decreased gradually with time of degradation. (**C**) The responsiveness of cells to rhKGF-1 and to the therapeutic cytokines rhEGF, rhEPO, NGF, and rhbFGF. (**D**). The responsiveness of cells to rhKGF-1 and rhKGF-2. RLU = Relative Luciferase Units. The mean ± SD is shown in each curve.

As mentioned in the ICH Q2 (R1), degradants are one of the typically investigation of specificity. Here, forced degradation of rhKGF-1 was induced through thermal stress. An rhKGF-1 sample was subjected to 37 °C for different days to induce specific forced degradation. Figure 3A,B summarized the results of specificity with the forced degradation study. Compared to an in-house rhKGF-1 reference and the untreated sample (0 day), the RLU of degraded rhKGF-1 (37 °C for 1 d, 3 d and 5 d) did not reach the top asymptote (Figure 3A). A gradual decrease in relative bioactivity was observed, from 0.98 of the untreated sample to 0.03 of the degraded rhKGF-1 for 5 days (Figure 3B).

Furthermore, the specificity of the new bioassay was elaborated in another assay. Four other therapeutic cytokines, i.e., rhEGF, rhEPO, rhbFGF, and NGF were treated with the rhKGF-1 bioactivity procedure, and we then determined the luciferase activity by adding to HEK293-Luc cells. The results demonstrated that HEK293-Luc cells responded to rhKGF-1 in a dose-dependent activity, but did not respond to rhEPO, rhbFGF, and NGF. For rhEGF, a slight curve appeared, but the RLU of the top asymptote was significantly lower than that for rhKGF-1 (Figure 3C). It is also worth highlighting that the HEK293-Luc cells responded to rhKGF-2 in a dose-dependent manner. This response to rhKGF-2 occurred at a higher initial concentration (300 ng/mL) and lower sensitivity (with an EC_50_ value of 6.28 ng/mL) than the response to rhKGF-1 (Figure 3D).

#### 2.3.2. Linearity

**Figure 4:**
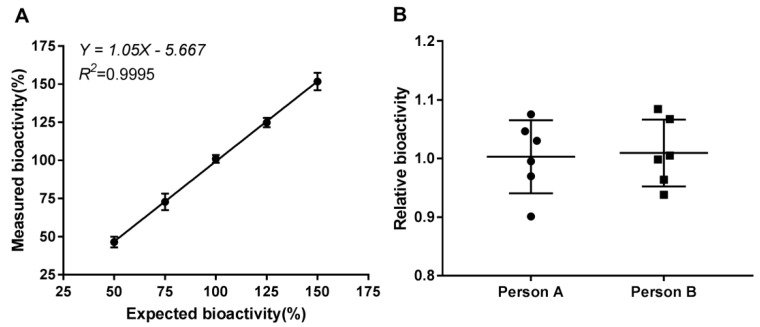
The results of the linearity and intermediate precision tests. (**A**) Linearity plot for the expected bioactivity against the measured bioactivity. Each point indicates the mean of three replicates. (**B**) Intermediate precision. Tests of six batches of rhKGF-1 were performed by two persons on different days. The mean ± SD and representative linear regression of four independent experiments is shown.

The linearity of an analytical method is its ability to give test results which are directly proportional to the concentration of analyte in the sample. For linearity validation, the percentage of relative bioactivity from five concentrations in the range of 50% to 150% of optimized initial concentration, i.e., 120 ng/mL, were determined by the EC_50_ value of an in-house reference at five different concentrations. The data were obtained three times per experiment in four independent experiments executed on four different days and were analyzed using a linear regression model. The CV of measured bioactivity for five different concentrations was calculated to be 0.44% to 4.96%, which was < 5% (Table S1). The measured bioactivity versus expected bioactivity indicated a good linearity (*R*^2^ > 0.9954 in each experiment), suggesting excellent linearity of the established method (Figure 4A).

#### 2.3.3. Accuracy

According to the ICH Q2 (R1) PART II, the accuracy of a method should be reported as the rate of recovery of a known added amount of analyte in a sample. So, we verified the percentage of an rhKGF-1 in-house reference recovered from our rhKGF-1 samples by analysis of three repeated assays each day on four different days. As shown in Table 1, satisfactory results for intra-day CV values for the final rhKGF-1 were obtained. These values ranged from 3.92% to 7.51%, and the inter-day CV was 6.91%. The average recovery rate was 92.75%, and the 95% confidence intervals (CI) of reference recovery were 88.68% to 96.82%. Thus, the acceptance recovery rate was satisfactory, and the method demonstrated sufficient accuracy.

#### 2.3.4. Precision

The precision of an analytical method may be investigated by the method’s repeatability (also termed intra-day precision) and intermediate precision, given as the CV. The repeatability was estimated from the results of relative bioactivity on four different days and in triplicate on each day for each sample. A batch of the final product and a bulk batch of rhKGF-1 were used for this purpose. As shown in Table 2, the maximum intra-day and inter-day CV values were lower than 5.00%. To assess the intra-plate precision, five repeated final product tests were performed in the same plate, and the resulting CV was 4.85% (data not shown).

To validate the variations within laboratories, intermediate precision was determined on different days by two operators [31]. Six different batches of rhKGF-1 (final products and bulk batches) were involved in this test. The mean relative bioactivities were 1.003 for person A and 1.009 for person B. Overall, the statistical indifference of the results suggested that the new bioassay was characterized by consistent performance and a good intermediate precision (Figure 4B).

#### 2.3.5. Stability of the HEK293-Luc Cell Line

**Figure 5:**
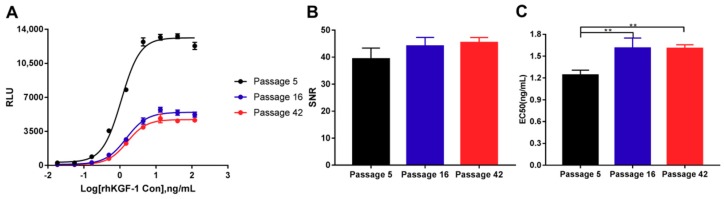
Stability of HEK293-Luc cell lines. (**A**) The responsiveness of HEK293-Luc cells to rhKGF-1 at different passages. (**B**) SNR of HEK293-Luc cells at three passages. (**C**) EC_50_ of HEK293-Luc cells at three passages. RLU = Relative Luciferase Units. SNR = Signal-Noise-Ratio. The mean ± SD is shown on each curve. ** *p* < 0.001.

The stability of the HEK293-Luc cell line and the response to rhKGF-1 are crucial for the cells to be used in the bioassay. Stability was evaluated by comparing EC_50_ value and SNR in response to rhKGF-1 at three different passages. We obtained parallel dose-response curves, indicating the consistency of the cell line between passage 5 and passage 42 (Figure 5A). The SNR showed a moderate tendency to increase with increasing cell passage number, from 39.35 at passage 5 to 45.39 at passage 42, but no significant difference was observed between three different passages (Figure 5B). However, the EC_50_ values associated with the sensitivities of passage 16 and passage 42 were significantly higher than that of passage 5, having changed from 1.24 to 1.61. Even so, the EC_50_ of passage 16 and passage 42 were the same as each other, 1.61 (Figure 5C). Therefore, the responsiveness of the HEK293-Luc cell line to rhKGF-1 was proven to be highly stable, especially between passage 16 and passage 42.

#### 2.3.6. Comparison of Responsiveness to rhKGF-1 between HEK293-Luc and HaCat-Luc Cell Lines

**Figure 6:**
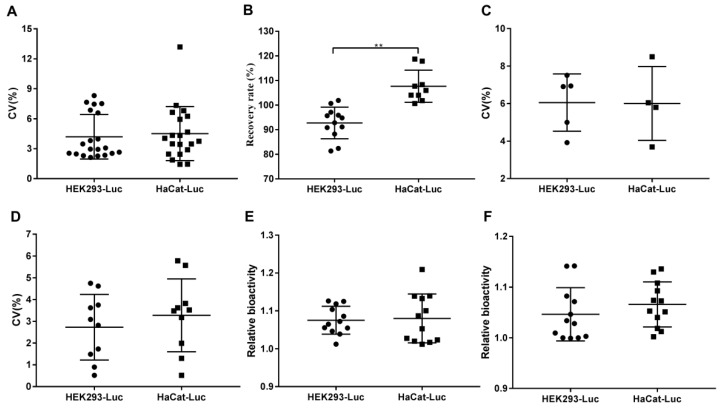
Comparison of validation between the HEK293-Luc and HaCat-Luc cell lines responding to rhKGF-1. Validation of bioactivity procedures is depicted in detail in the Results section. (**A**) CV of linearity validation (n = 20). (**B**) Recovery rate (n = 12 for the HEK293-Luc cell line and n = 9 for the HaCat-Luc cell line), *p =* 0.0057. (**C**) CV of accuracy validation (n = 5 for the HEK293-Luc cell line and n = 4 for the HaCat-Luc cell line). (**D**) CV of precision validation (n = 10). (**E**) Relative bioactivity of the final rhKGF-1 product (n = 12). (**F**) Relative bioactivity of bulk rhKGF-1 (n = 12). The mean ± SD is shown. ** *p* < 0.01.

As shown in Figure 6 and Table S2, the CV values of method validation, recovery rates, and relative bioactivity were compared between HEK293-Luc and HaCat-Luc cell lines responding to rhKGF-1. Concordance between HEK293-Luc and HaCat-Luc cell lines was demonstrated with the CV values of the linearity (Figure 6A), accuracy (Figure 6C), and precision (Figure 6D). In comparing the recovery rates, the HaCat-Luc cell line showed a significantly higher rate (107.70%) than did the HEK293-Luc cell line (92.75%) (Figure 6B). Additionally, equivalent relative bioactivity with final rhKGF-1 and bulk rhKGF-1 was observed between the two cell lines (Figure 6E,F). In summary, when considering the concordance levels, only the recovery rate had a difference, whereas the CV and relative bioactivity supported a conclusion of greater agreement between the HEK293-Luc and HaCat-Luc cell lines.

## 3. Discussion

KGF is a member of the FGF family and is an epithelial-specific growth factor. Studies have indicated that KGF can induce cell proliferation and mitogenic responses in various cell types by binding to the KGF receptor (KGFR) [3]. The KGFR is cell surface FGFR2 IIIb, a splice variant of the FGFR2 gene that belongs to the receptor tyrosine kinase (RTK) family [11]. *KGFR* mRNA has been detected in almost all of the examined tissues, yet KGFR is expressed exclusively in epithelial cells. It confers the characteristics of proliferation and differentiation with KGF stimulation [2,13]. A previous study indicated that *Fgfr2* gene expression in embryonic kidney tissue was one of the most relevant aspects of renal development [32]. Therefore, we proposed HEK293 and HaCat cell lines as candidates in the subsequent study, as the former was derived from human embryonic kidney, and the HaCat cell line was derived from a human keratinocyte cell line spontaneously immortalized from a primary culture of keratinocytes, and widely used as a model to study keratinocyte differentiation [33,34]. Furthermore, the HEK293 and HaCat cell lines are robust enough to be handled and cultured.

FGFR2 IIIb plays a critical role in the MAPK, PI3K/AKT/mTOR, and PLCγ intracellular signaling pathways, and it contributes to the biological activity of KGF involved in proliferation, differentiation, and migration [3,35]. In the MAPK pathway, the KGF-KGFR complex induces autophosphorylation of KGFR’s tyrosine kinase domains and phosphorylation of its intracellular domain. These changes assemblages opportunities for FGFR substrate 2α (frs2α) docking protein, which then sequentially activates Raf, MEK, and ERK. Finally, the activated ERK translocates to the nucleus, where it activates transcription factors and induces cell proliferation [14,36]. In this study, we suggest that SRE response element can drive expression of the luciferase reporter gene in response to activation of the MAPK/ERK signaling pathway. Furthermore, we describe a new bioassay to determine the bioactivity of rhKGF-1 based on the luciferase reporter gene driven by SRE in HEK293 and HaCat cells bearing FGFR2 IIIb. The two target cell lines were labeled with HEK293-Luc and HaCat-Luc.

It is well known that heparan sulfate (HS) in the extracellular matrix and proteoglycans on the surfaces of cells act as obligatory co-receptors, facilitating the binding of FGF to FGFR. This confers FGF dimerization, increases receptor binding affinity, and stabilizes the FGF-FGFR complex [21,25,36,37]. Moreover, KGF-1 and KGF-2 have been shown to bind to HS. In the optimized procedure, heparin, a proxy HS, was investigated in assay media. We found that the RLU value of top asymptote of 0–2 μg/mL of heparin was higher than that of 5–40 μg/mL of heparin, and a better SNR was appeared in 2 μg/mL of heparin in assay media.

The method validation was in line accordance with the regular requirements as stated in the ICH Q2 (R1) guidelines, AAPS/FDA Bioanalytical Workshop and the Chinese Pharmacopoeia. Typical validation which should be considered are specificity, linearity, accuracy, precision, and robustness [38,39]. The new bioassay described was based on double-transfected cells harboring the full-length FGFR2 IIIb and luciferase-SRE, which were obtained from Promega with an improved synthetically derived luciferase reporter gene (luc2P). First we found that the validated method produced a better SNR and sensitivity than cells without the FGFR2 IIIb but having the luciferase-SRE vector (data not shown). Second, the specificity was assessed using other therapeutic cytokines (Figure 3C). We found that rhKGF-1 appeared to exhibit absolute specificity for FGFR2 IIIb, whereas rhKGF-2 exhibits similar ability to bind to FGFR2 IIIb but also binds FGFR1 IIIb [2]. We think that a slight responsiveness to rhEGF is probably due to the same signaling pathway as seen in KGF, and HEK293 cells expressing EGF receptor. Therefore, to improve responsiveness to KGF, specific FGFR2 IIIb was stably transfected into cells. Non-responsiveness to rhbFGF (rhFGF2) was seen, which was the expected result, because its specific receptors, i.e., FGFR1c, FGFR3c, FGFR2c, FGFR1b, and FGFR4, are different from FGFR2 IIIb [9]. This also showed that there is high sensitivity in detecting the bioactivity of degraded rhKGF-1.

As for the acceptance criteria of CV values in analytical method validation, we followed the AAPS/FDA Bioanalytical Workshop acceptance criteria for precision and accuracy, i.e., the acknowledged CV values of 15% to 20% [40]. Actually, in our study, all of the CV values had an advantage over the acceptance criteria. Thereinto, the CV values of repeatability and linearity were less than 5%; the CV values of accuracy were lower than 8.00%. Additionally, a clear recovery rates and stability for desired HEK293-Luc cells were demonstrated, as the recovery rates ranged from 81.35% to 101.90%, and there was stability of the sensitivity and SNR between passage 16 and passage 42.

We have optimized and characterized two cell lines to measure the bioactivity of rhKGF-1. Although concordance between HEK293-Luc and HaCat-Luc cells (except in the recovery rate) was shown, the HEK293-Luc cell line was preferentially chosen compared to HaCat-Luc cell line because of its increased sensitivity and a higher SNR to rhKGF-1 treatment. Regarding the recovery rate, it is not essential to have 100% recovery, but it is important that the recovery be reproducible [41]. Moreover, the parallelism of the dose-response curves and the similarity of SNR and EC_50_ between RPMI 1640 media and cell culture plate obtained from Thermo Fisher indicated the consistency and robustness of the HEK293-Luc cell line in determining the bioactivity of rhKGF-1 (Figure S2B).

## 4. Materials and Methods

### 4.1. Cells and Materials

The HEK293 cell line (CRL-1573™) was obtained from the American Type Culture Collection (Manassas, VA, USA). The HaCat cell line (3111C0001CCC00037) was purchased from National Infrastructure of Cell Line Resource (Beijing, China). All of the cells were maintained in DMEM containing 10% fetal bovine serum (FBS) at 37 °C in a humidified 5% CO_2_ incubator. DMEM, RPMI1640, FBS, puromycin, and hygromycin B were purchased from Gibco (Grand Island, NY, USA). pGL4.33[luc2p/SRE/Hygro] firefly luciferase reporter plasmid and ViaFect™ transfection reagent were obtained from Promega (Madison, WI, USA). Lentivirus production for the human FGFR2 IIIb gene (GenBank No: NM_022970) was completed by the Genechem Company (Shanghai, China). The Britelite Plus Reporter Gene Assay System was obtained from PerkinElmer (Waltham, MA, USA). An in-house rhKGF reference, rhKGF1, rhKGF2, recombinant human epidermal growth factor (rhEGF), recombinant human basic fibroblast growth factor (rhbFGF), recombinant human erythropoietin (rhEPO), and nerve growth factor (NGF) were archived therapeutic drugs that had been preserved at 4 °C or −80 °C in our laboratory.

### 4.2. Preparation of Desired Responsive Cells to rhKGF-1

The serum response element (SRE)-luciferase reporter plasmid was transfected into exponentially dividing HEK293 and HaCat cells using ViaFect™ transfection reagent according to the manufacturer’s instructions. The transfected cells received regular changes of DMEM-10% FBS with hygromycin B (300 μg/mL), and were continuously cultured for 3–4 weeks. For FGFR2 IIIb, hygromycin B-resistant HEK293 and HaCat cells were then infected with lentivirus containing the human FGFR2 IIIb gene as per the manufacturer’s recommendations. Following a change of media with hygromycin B and puromycin (3 μg/mL), the two cell lines were incubated for an additional 72–120 h. Then, a clonal cell line derived from a single cell was produced by limiting dilution in a 96-well plate, using 0.8 cells per well from the stably transfected cells. After isolating the clones, clone scale-up and screening assessments (responsive to rhKGF-1 stimulation) were performed. We desired cells that would be highly responsive to rhKGF-1, and obtained such cells and named them HEK293-Luc and HaCat-Luc. The target cell lines were maintained in DMEM-10% FBS with 200 μg/mL of hygromycin B and 1.5 μg/mL puromycin. Hereafter this media is referred to as growth media.

### 4.3. Bioactivity Assay

A cell-based bioassay was performed as described previously with moderate modifications [29,30]. In brief, 4 × 10^4^ cells in 60 μL assay media (DMEM with 0.5% FBS and 2 μg/mL heparin) were added to each well of a 96-well cell plate (3903, Costar, New York, NY, USA) and were incubated for 16–18 h in a humidified 5% CO_2_ incubator at 37 °C.

An in-house rhKGF-1 reference and rhKGF-1 were diluted by serial 3-fold dilutions with assay media, starting from initial concentrations of 240 ng/mL on the HEK293-Luc cells and 600 ng/mL on the HaCat-Luc cells. Then, 60 µL of serially diluted rhKGF-1 was added to each well. It should be noted that the final concentrations of rhKGF-1 were 120 ng/mL for HEK293-Luc and 300 ng/mL for HaCat-Luc. After incubation for 4–5 h at 37 °C, with 5% CO_2_, the supernatant was removed from each well, followed by addition of 60 µL Britelite Plus Reporter Gene Assay reagent. After 5 min of incubation at room temperature in the dark, the luciferase activity was determined by a Luminoscan Ascent plate reader (SpectraMax M5, Molecular Devices, San Jose, CA, USA).

### 4.4. Preparation of Forced Degradation from rhKGF-1

The specificity of the bioassay was assessed by the presence of degraded components of rhKGF-1. It is known that with increasing temperature, proteins may undergo conformational changes, subsequently leading to other degradation reactions [42]. Therefore, forced degradation of rhKGF-1 was induced through thermal stress. That is, the reconstitution of freeze-dried rhKGF-1 was incubated at 37 °C for 1 d, 3 d, and 5 d. The relative bioactivity for the stressed samples was compared with the bioactivity of samples that had not undergone degradation treatment. We performed this bioactivity assay using HEK293-Luc cells under the same conditions as described above.

### 4.5. Data Analysis and Statistics

All of the statistical analyses were performed using SoftMaxPro (Molecular Devices) and GraphPad Prism 7.0 (GraphPad Software Inc., San Diego, CA, USA). The sigmoidal curve and the concentration for 50% maximal effect (EC_50_) were calculated through a four-parameter model (dose-response-stimulation). The relative bioactivity of rhKGF-1 is shown as the ratio of the EC_50_ values of an in-house reference to the EC_50_ values of the samples. The SNR is indicated by the ratio of the top asymptote to the bottom asymptote. Comparisons between two groups were performed using a two-tailed Mann-Whitney test, and multiple comparisons were performed using a Kruskal-Wallis test with Dunn’s multiple comparisons. *p*-values < 0.05 were deemed to be statistically significant.

## 5. Conclusions

In summary, we describe a highly timesaving, sensitive, and simple bioassay. This is the first use of an SRE-dependent reporter gene assay to determine rhKGF bioactivity. This bioassay has a superior specificity, linearity, accuracy, precision, and robustness, and could provide invaluable information for quality control during the manufacture, research, and development of therapeutic rhKGF.

## Figures and Tables

**Table 1:** The recovery rate in the final rhKGF-1 sample.

	Recovery Rate (%)			Intra-day CV (%)	Mean	SD
	1	2	3			
1	97.13	91.17	100.70	5.00	96.33	4.82
2	94.80	95.90	101.90	3.92	97.53	3.82
3	82.40	88.27	95.70	7.51	88.79	6.67
4	92.82	90.86	81.35	6.94	88.34	6.13
Inter-day CV (%)	6.91					
Mean	92.75					
SD	6.41					

**Table 2:** The repeatability of final and bulk rhKGF-1 samples.

Sample	Intra-day CV (%)				Inter-day CV (%)	95% CI of Relative Bioactivity
	1	2	3	4		
rhKGF-1	1.49	3.09	1.73	3.75	2.82	1.05–1.10
rhKGF-1 bulk	4.62	0.90	3.62	0.52	4.75	1.01–1.08

## Supplementary Materials

The following are available online, Figure S1: The establishment of responsive HaCat-Luc cells for rhKGF-1 bioactivity, Figure S2: The robustness of assays on rhKGF-1 bioactivity, Figure S3: Determination of the quantitation range of rhKGF-1, Table S1: Statistical evaluation of linearity studies, Table S2: Statistical data of linearity, recovery and precision studies between HEK293-Luc and HaCat-Luc cell lines.
