# Supplementary material for: Bioactivity Determination of a Therapeutic Recombinant Human Keratinocyte Growth Factor by a Validated Cell-based Bioassay
Source: Molecules. 2019 Feb 15;24(4):699. doi: 10.3390/molecules24040699 (PMC6412437; doi:10.3390/molecules24040699)
Supplement: Supplementary file 1 [file molecules-24-00699-s001.pdf]

Table S1 Statistical evaluation of linearity studies

Expected bioactivity	50% of target	75% of target	100% of target	125% of target	150% of target
Measured bioactivity (%)	47.01	72.58	101.42	124.69	149.97
CV (%)	4.96	3.66	0.44	0.83	1.61

Table S2 Statistical data of linearity, recovery and precision studies between HEK293-Luc and HaCat-Luc cell lines

	HEK293-Luc	HaCat-Luc	P value
CV of linearity validation (%)	4.20	4.51	0.7584
Mean recovery rate (%)	92.75	107.70	0.0057
CV of accuracy validation (%)	6.06	6.01	0.9048
CV of precision validation (%)	2.73	3.28	0.5165
Relative bioactivity of final rhKGF-1 product	1.08	1.08	0.8197
Relative bioactivity of bulk rhKGF-1	1.05	1.07	0.2136

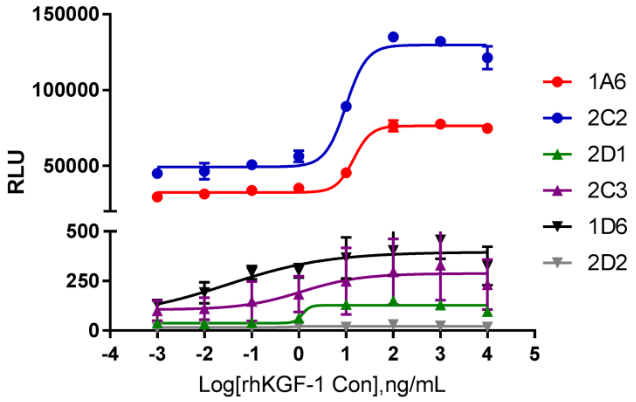

Figure S1 The establishment of responsive HaCat-Luc cells for rhKGF-1 bioactivity

The six clones from single-cell dilutions of HaCat-Luc cells bearing SRE-luciferase and human KGFR2 IIIb were evaluated by luciferase activity with rhKGF-1 stimulation (initial concentration of 10,000ng/mL, dilution ratio of 1:10). The curves were calculated in a four-parameter model. RLU, Relative Luciferase Units. The mean ± SD is shown on each curve.

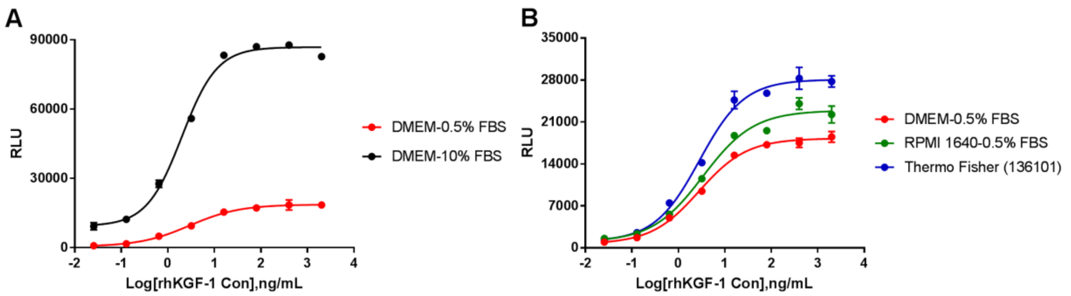

Figure S2 The robustness of assays on rhKGF-1 bioactivity

The luciferase activity was determined after stimulation of rhKGF-1 diluted with different assay media (initial concentration of 2,000 ng/mL, dilution ratio of 1:5). A. The impact of different FBS concentrations. B. The impact of RPMI 1640 cell culture media and cell culture plate obtained from Thermo Fisher. The curves were calculated in a four-parameter model. RLU, Relative Luciferase Units. The mean ± SD is shown on each curve.

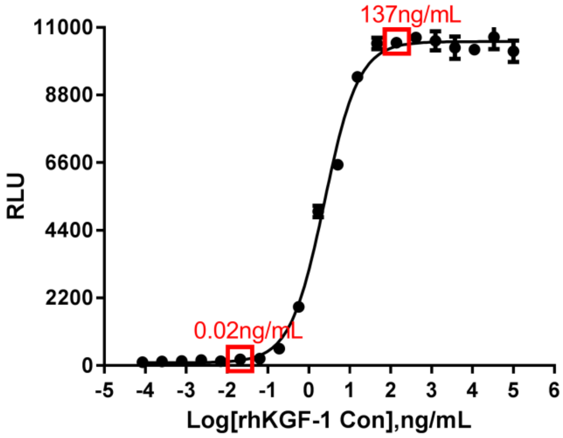

Figure S3 Determination of the quantitation range of rhKGF-1

The quantitation range of rhKGF-1 was determined by luciferase activity induced by rhKGF-1 (initial concentration of 100,000 ng/mL, dilution ratio of 1:3). The curves were calculated in a four-parameter model. RLU, Relative Luciferase Units. The mean ± SD is shown. Red squares indicating the working concentrations are close to the top and bottom asymptotes.
